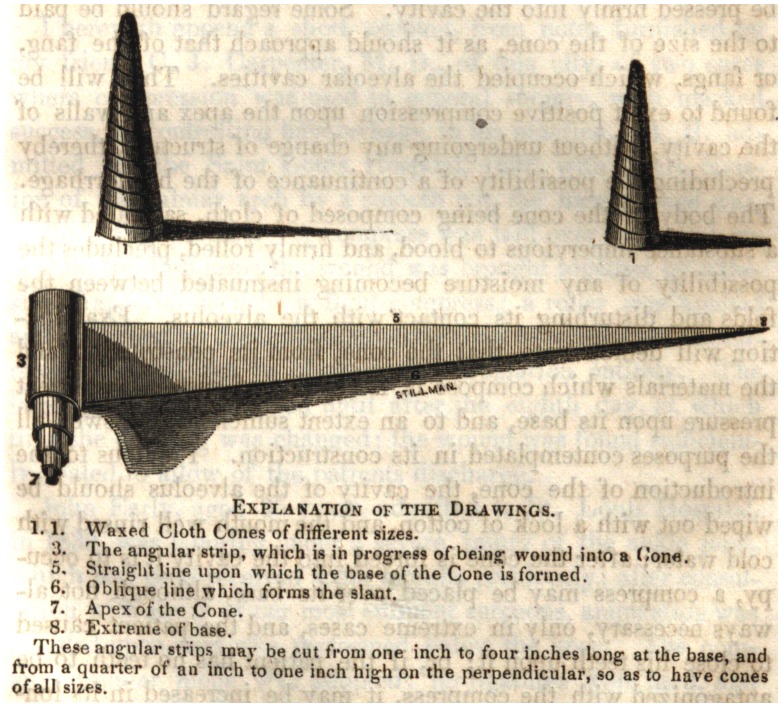# Observations on Hæmorrhage

**Published:** 1848-10

**Authors:** B. B. Brown

**Affiliations:** St. Louis, Mo.


### OBSERVATIONS ON HÆMORRHAGE,

READ *before the Mississippi Valley Association of Dental Surgeons, at Cincinnati, Ohio, September 6th,* 1848,

BY B. B. BROWN, M. D., D. D. S.,
*of St. Louis, Mo.*

GENTLEMEN:

As I had the honor to be one of the number designated at our last annual meeting, to read a paper before you on the present occasion, I shall, therefore, present, for the consideration of the dental profession, in a brief, and, I regret to say, imperfect manner, some observations on Alveolar Hæmorrhage.—This subject has not been elaborately treated in any of the works on Dental Surgery, and yet, amongst the vast number of evils which beset man, and tend to shorten his mortal career, there is *none* more alarming in its progress, or certainly fatal in its results, than uncontrolled hæmorrhage; and none, coming under the purview of the profession, demands, in a greater degree, a varied combination of acquirements in order to arrest its progress successfully, and to terminate the serious apprehensions of the patient. Cases of alarming hæmorrhage, consequent upon the operation of extraction of the teeth, are fortunately rare; yet, as they *have* occurred, and *have*, occasionally, terminated fatally, they *may* occur again; hence, the accomplished practitioner will frequently find his best judgment comparatively at fault, in procuring a favorable conclusion to his efforts, in applying remedies for that purpose. With the view to a more complete and comprehensive understanding of this subject, let us proceed to consider, briefly, the anatomical structure of the arterial system which relates to those parts, and I hope the society will pardon me for thus trespassing upon the time and patience of the members, in the examination of a matter, which, I doubt not, is perfectly familiar to all, and which has often received their scrutinizing attention; yet, perhaps some stray thought, or casual suggestion, advanced in this paper, may be of value to some member of the profession; in which case the objects of its preparation will have been fully accomplished.

The arterial system is composed of a congeries of vessels which arise from the heart, and distribute the blood to all parts of the body. Each artery, according to Bichat, (whom I adopt as authority,) is composed of three tunics, or coats; to-wit:

*First*, an external coat or fibro-cellular membrane, delicate, tolerably dense, and forming a constituent part of the arterial tube. In the great arteries this coat is divided into two laminæ, one external, which approximates to cellular texture; the other internal, yellowish, and tough, which resembles the layers of the middle coat. Its texture is composed of fibres, interlaced and oblique, more separated without than within, which separation is especially apparent in the distension of the artery, either longitudinally, or circularly. The small arteries have this coat thicker than the large ones in proportion to their size. In this membrane are inserted many soft and extensible filaments which come from a species of sheath proceeding from the surrounding cellular texture, that covers the artery. By means of this arrangement, the artery slides easily in the interior of this cellular canal, and the retraction of divided arteries is very much facilitated by it, and this is the only relation which exists between the artery and the cellular texture.

*Second*, the middle coat, or membrane, of the arteries is firm, compact, very apparent in the great arteries, but less evident as they decrease in size, and in the very small ones it becomes insensibly lost. This membrane is composed of very distinct fibres adhering to each other, easily separated, however, and arranged in layers in such a manner, that, after having raised the cellular covering, we can, without difficulty, separate these different layers from each other. Most anatomists consider the fibres found in this coat muscular, but the muscular tissue is soft, loose, and very extensible, while this coat, on the contrary, is firm and solid, breaking before it yields. From the effect of various re-agents upon it, Bichat placed it where it properly belongs, to-wit: among the fibrous membranes composed of the yellow elastic fibrous tissue.

The *third*, or internal coat, sometimes called the serous, and at others the nervous coat, is smooth and of a uniform texture like the serous membranes, as is very apparent by holding it up to the light. It differs from them, however, by a kind of brittleness that characterizes it, as it is easily broken and torn, on the least effort in the circular direction, but resists the application of considerable force when applied in the longitudinal direction. The arteries are supplied with arteries, (vassa vassorum,) veins, and nerves, and consequently, are exposed to diseases as well as the other parts of the system; with these diseases we have nothing to do at present, except so far as they tend to produce hæmorrhage.

The mouth is abundantly supplied with blood by means of vessels which are branches of the external carotids. The right carotid artery, which arises from the arteria innominata, is shorter and larger than the left, which comes from the arch of the aorta. The common carotid artery ascends the side of the neck to a level with the upper border of the thyroid cartilage, where it divides into the external, and internal, carotids. The external carotid ascends the neck perpendicularly, from the upper border of the thyroid cartilage to the space between the neck of the inferior maxillary bone, and the meatus auditorious externus.

The lingual artery, being the second branch of the external carotid, crosses the great corner of the os hyoides, and running parallel with its upper border, it ascends to the under surface of the tongue, and running forward, in a serpentine direction, terminates at the tip of the tongue, under the name of the varine artery. The facial artery, which is the third branch of the external carotid, first gives off the inferior palatine artery, and is distributed to the tonsils and soft palate. The submental, or third branch of the facial, forms an anastamoses with branches of the sublinguel and inferior dental arteries. The inferior labial, or fifth branch of the facial, is distributed to the muscles and integuments of the lower lip. The inferior coronary, or sixth branch of the facial, runs along the edge of the lower lip close to the mucous membrane, and inosculates with the corresponding artery of the opposite side.

The superior coronary, or seventh branch of the facial, runs along the upper lip, inosculating with the opposite superior coronary. The internal maxillary artery passes inwards, behind the neck of the lower jaw to the deep structures of the face; it gives off, as its second branch, the inferior dental, which descends to the lower jaw, and entering the dental foramen, in company with the dental nerve, distributes branches to the teeth, and passing through the mental foramen, it inosculates with the inferior labial and submental branches of the facial.—The superior dental, or tenth branch of the internal maxillary, passes down upon the surface of the superior maxillary bone, and sends its branches through several small foramina, to supply the posterior teeth of the upper jaw. The infra-orbital, or eleventh branch, runs along the infra-orbital canal, sending down branches to the mucous lining of the antrum, and teeth of the upper jaw, and, escaping from the infra-orbital foramen, inosculates with the facial and transverse facial arteries.—*Wilson's Anat.*

"Hæmorrhage is said to be active, when there is a preternatural flow of blood to the part, attended with an increased vascular excitement. The passive hæmorrhage depends either on mere relaxation and inactivity of the vessels, without any morbid changes in the constitution of the blood in consequence of previous disease, excessive discharges of all kinds, or other exhaling influences, or it is connected, and probably in a greater degree dependent on a thin watery, or dissolved state of the blood; and therefore, incapable of communicating healthy impressions to the general and capillary system of vessels."*

Bichat says, "If I should class hæmorrhage, I should distinguish them, *first*, into those that come from exhalation; *second*, into those that are produced by rupture. I should place among the first, the bloody sweats, the mucous, serous, cellular hæmorrhage, &c., &c. Among the second, those that accompany wounds, aneurisms, &c."† "Blood, from whatever organ it flows, may have two causes for its issue. The vessels may be ruptured by a morbid distension and impetus; or they may give way from debility and relaxation, their tunics breaking without any peculiar force urged against them, or their exhalants, admitting the flow of red blood instead of the more attenuate serum."‡ There are numerous local causes operating in the production of hæmorrhage. Ossification of the arteries, by causing ulceration of their coats, may produce it. A blow upon the head may give rise to fatal hæmorrhage by the rupture of an artery. The slough that is cast off from a gunshot wound may implicate an artery and cause very serious, if not fatal, secondary hæmorrhage.—*Gibson's Surgery.*

"Extensive ill-conditioned ulcers, by penetrating deeply, and laying waste the soft parts, may occasion fatal hæmorrhage by opening large arteries."‖ Hæmorrhage may arise in ulcers, either from the increased action which produces an hæmorrhagic tendency in the new formed vessels of the part, or bleeding may occur from the complete relaxation, or weakness of the vessels."§ A wound of the soft parts may implicate an artery and cause hæmorrhage. Scorbutus, or scurvy, predisposes to hæmorrhage.

From slight wounds serious hæmorrhage, and even death, may follow. Such cases are said to be complicated with an hæmorrhagic diathesis.

---

*Eberle's Practice of Med., vol. 1., pp. 538-9.

†Bichat's General Anat. (translated by Hayword,) vol. 2, p. 82.

‡Good's Study of Medicine, vol. 2, p. 456.

‖Gibson's Surgery. vol. 2, p. 66.

§Abernethy, (by Castle.)

Hæmorrhage may occur from the nose, constituting that variety known as epistoxis; when it occurs from the lungs it is denominated hæmoptysis; that from the stomach is called hæmatemesis; and that from the kidnies is known by the name of hæmaturia, while that from the cavity of the rectum, or verge of the anus, usually proceeds from hæmorrhoids.

Inordinate alveolar hæmorrhage sometimes follows the operation of extracting a tooth, and requires professional aid to arrest it; this condition generally depends upon some of the causes already enumerated, but it is not unfrequently accelerated by, and is sometimes dependent upon the imprudence of the patient in sucking the bleeding alveolus, thereby preventing the formation of a coagulum in its cavity, the means set up by nature to arrest hæmorrhage. The patient, it is true, does this involuntarily; the novelty, warmth, and saline taste of the blood, tend to beguile him into the error which he is committing. It is rare that a patient will ever acknowledge any participation, or agency *on his part*, in bringing about such a condition; however, the mischief is soon accomplished, while the lacerated end of the bleeding vessel lays flaccid in its bony canal, its contractile power greatly diminished, or entirely lost; hence, the hæmorrhage must continue unabated. A high state of inflammation in the soft parts surrounding a tooth, brought about by inflammation of the periostum of the fang, and subsequent suppuration of that tissue, excites the absorbents to take up sufficient osseus matter for the accommodation of the sac pressing upon the bony structure of the jaw. This sac, not unfrequently, lays bare the main trunk of the artery which gives of its delicate twigs to supply the teeth with blood; hence, the presence of continued inflammation would produce increased vascular action, and consequently, a determination of blood to the part. The extraction of a tooth under such circumstances, (and generally it is the only alternative,) with, or without, imprudence on the part of the patient, is often followed by alarming hæmorrhage. Injuries of the jaws, combined with fracture, may wound vessels traversing their bony structure, and produce troublesome hæmorrhage. The operation of extraction may be performed under the most favorable circumstances, and yet alarming hæmorrhage follow, without any agency or indiscretion on the part of the patient, wherever a hæmorrhagic diathesis may exist.

When hæmorrhage proceeds from an artery, the blood is of a bright scarlet color, and is ejected from the vessels by jets, jerks *or per saltum,* as it has been denominated; if it proceeds from the veins, it is of a dark purple or red color, and flows in an unbroken stream.

TREATMENT—"The general indications to be kept in view in the treatment of hæmorrhages, are: 1. To lessen the momentum of the circulation if it be above, or at its natural standard; 2. To diminish the determination of blood to, and moderate the local vascular action in, the part from which the hæmorrhage occurs; and 3. To excite a contraction of the vessels of the part. The first indication is to be fulfilled by venesection and the exhibition of sedatives—as nitre, digitalis, cold, &c. The second indication demands counter-irritating and revulsive applications —such as cold, applied, if practicable, to the part from which the blood flows, and blisters, sinapisms, warmth, and rubefacient frictions, on remote situations. The last indication requires the internal use of astringents, such as sugar of lead, alum, muriated tincture of iron, &c., and when the situation of the part will admit of it, the external application of styptics."*

Dr. Goddard remarks, "that a hæmorrhagic diathesis, or tendency to shed blood from slight injuries, is very frequently hereditary, and lasts for life; while at others it is merely temporary, and is the effect of long continued ill-health, or a state of the system brought about by unfavorable circumstances, such as confinement, bad diet, salt provisions, &c., &c. In those persons in whom this predisposition is hereditary, the slightest injury will cause very great, and sometimes fatal, loss of blood, and this condition is very difficult to remedy. When the dentist is aware of its existence, no consideration should induce

---

*Eberle's Practice of Medicine, vol. 1. p. 544.

him to remove a tooth, as the death of his patient might follow the operation."*

The following case of hæmorrhage, combined with a hæmorrhagic diathesis, will serve to illustrate, in a remarkable degree, the subject under discussion. In the fall of 1839, I was called to see a young man, aged 19 years, who had had the second bicuspid tooth of the superior jaw extracted, seven days previously; he was, at the time, laboring under preternatural alveolar hæmorrhage, which had continued, with but little cessation, from the time the operation was performed until I was called in. His pulse was below the natural standard, skin sallow, and features of the face exhibiting much anxiety. I made the inquiry if he did not bleed inordinately from receiving slight injuries; he replied, that he generally bled a *week* from the scratch of a pin. He was considerably weakened by the loss of blood, notwithstanding a great variety of remedies had been employed to arrest the bleeding, by the dentist who extracted the tooth, and who had abandoned the case in despair, at the time it fell into my hands. I immediately cleared away the blood, and caused the mouth to be rinsed out with a cold saturated solution of salt water, (muriate of soda,) and applied a "*waxed cloth cone*," which instantly arrested the hæmorrhage from the alveolar cavity. But it was now discovered that the surrounding gum, where no wound existed, was giving off blood; and this remarkable condition continued to spread until the whole mucous membrane appeared to be involved; so that, when the mouth was opened, its surface could be seen covered with stalactitical coagula pendant from its roof and walls. Acetate of lead, combined with opium, was administered internally, warm stimulating applications to the extremities, and cold to the neck, face, and head; compresses of *waxed cloth* to the mouth, filling it up from time to time, in conjunction with the following lotions, to-wit: decoction of nut galls with sulph. aluminæ, solution of quinine, solution of sulphate of copper, ice water, &c., &c. On the afternoon of the second day, the hæmorrhage had evidently

*Goddard on the Teeth, p. 112.

diminished, but the patient was sinking, and from apparent symptoms, the lead had been exhibted as far as it was consistent with prudence. As the bowels were costive, I administered sulphate of soda, until free evacuations were produced; at the same time, I also abandoned the use of the lotions and compresses, and employed the *Oil of Ergot*, (secale cornutum,) which I prepared in the mean time, as a local application to the mouth by dipping locks of cotton in it, and laying them on the mucous membrane. This change of treatment was attended with entire success; for in a few hours, I had the satisfaction to arrest the most fearful hæmorrhage which it has been my lot ever to witness, as it had continued, with but slight intermission, for a period exceeding nine days. By subsequent treatment, a restoration of the young man to health was accomplished.

The oil of ergot (secale cornutum) was highly extolled, as a styptic remedy in arresting uterine hæmorrhage, some years ago, by a French writer. It is prepared by bruising the ergot in a mortar, and digesting it in sulphuric ether about twelve hours or longer; filter through paper, and place a wide, shallow glass vessel under the drop; in a short time the ether will evaporate and the residue will be the oil. I regard this substance as possessing remarkable properties, and invaluable in such cases as the one which I have just cited.

Goddard on the Teeth, page 115, recommends the following combination:—

| | | |
|---|---|---|
| ℞ | Sulphate of Soda, - - - - | 1℥ |
| | Muriate of Soda, - - - - - | ½℥ |
| | Chlorate of Potash, - - - - | 9ʒ |

Mix and divide into six parts; one of which may be administered every hour or two, until free purging is produced. The remedy diminishes the amount of serum, by the free watery purging which it produces, and the portion absorbed and mixed with the blood tends to confer a power of forming a firmer clot not possessed before."

A case is reported in the Am. Journal of Dental Science, vol. 6, page 320, from the Manchester Medical Times, in which there had been a hæmorrhage of ten days duration, following the extraction of a molar tooth; all local applications which the surgical attendants resorted to, having failed, the patient was put upon the internal use of the acetate of lead, and after this course had been continued for some time, sulphate of soda was administered with decidedly beneficial results.

A remarkable case of spontaneous hæmorrhage from the gums is mentioned in the 2d vol., no. 4, of the New York Dental Recorder, and copied into the 3d no., vol. 1, of the Register.—The bleeding continued upwards of three days, and resisted styptics and the actual cautery. When, finally, it was arrested by compresses of cotton, saturated with tincture of nut-galls, forced between the teeth where the seat of the hæmorrhage seemed to be.

After having premised the foregoing remarks upon constitutional hæmorrhage, let us now proceed to consider the treatment of the local form. The ligature and compression are the means generally adopted by surgeons of the present day to restrain local hæmorrhages, but, in addition thereto, there are many substances in use, which are intended to effect the same purposes, such as alum, kino, muriated tincture of iron, the mineral acids, mateco, nitrate of silver, sulphate of copper, kreosote, eau brocchieri, nut-galls, the actual cautery, &c., &c.

Celsus recommended a wound pouring out blood, to be filled with dry lint, over which should be placed a sponge wetted with cold water, or lint saturated with vinegar and water, and pressed on the part with the hand. But the principal reliance of the ancients, was upon the application of the actual cautery to the wounded vessel and the surrounding soft parts; the heat thus applied produced an eschar, which closed up the orifice of the vessel, and prevented the flow of blood. Secondary hæmorrhage, however, frequently resulted upon detachment of the eschars, and rendered the case more difficult than it had been before the application of the cautery was made. The reason of this is apparent; for when the eschar formed, a portion of the sound vessel was necessarily included in it, and upon the separation, an orifice larger than the former one was presented, and consequently, a more profuse hæmorrhage followed. Le Oran recommends the application of a button of alum, or vitriol, which he affirms will prevent, or arrest, hæmorrhage, if correctly applied, and confined to the extremity of the bleeding vessel. But styptics generally, have now given way to compression and the ligature; the ligature itself acts upon the principle of compression when it is applied to an artery.

"From the numerous and diversified experiments of Dr. Jones and others, it appears that a ligature, when applied to an artery with sufficient force, divides the internal and middle coats, leaving the external coat entire. The blood is arrested in its progress by the approximation of the sides of the vessel, soon coagulates and forms a plug, extending as high as the first collateral branch. This serves as a temporary barrier, and takes off the force of the circulation, from the ligature and the extremity of the artery; in the meantime, the divided edges of the artery pours out lymph, which is not only effused in the cavity of the vessel, but between its coats; the irritation, also, excited by the ligature, gives rise to an accumulation of lymph on the outer surface of the artery. At last the external coat, continually irritated by the ligature, sloughs or ulcerates, and the ligature is detached, leaving the mouth and edges of the vessels filled and surrounded by a bed of lymph, into which the vessels shoot, and by uniting the sides of the artery, form a permanent closure.—After a time the coagulum is absorbed and the channel of the artery, as high as the first anastamosing branch, is obliterated and converted into a solid cord; the circulation is maintained by the enlargement of the collateral vessels."*

But there are some cases in which a liagture cannot be applied to the bleeding vessels, as in cases of hæmorrhage from deep seated wounds, as those of the palmar arch, and that which takes place, sometimes, from the alveolus after the extraction of a tooth; in such cases we must rely upon the judicious application of well directed pressure. As various means have been proposed for arresting alveolar hæmorrhage, it may not be wholly uninteresting to examine some of the leading appliances recommended.

---

*Gibson's Surgery, vol. 2. p. 75.

Bell on the Teeth, page 307, recommends compression by means of lint firmly pressed into the bleeding alveolus. Gaviot on the diseases of the Mouth, page 144, advises cold and ascidulated gargles, compression by means of cotton, agaric, &c., dipped in acid, powdered resin, or gum arabic, to be applied to the part; also the actual or potential cautery. Snell on the Teeth, page 123, advocates, and recommends, the same treatment as pursued by Bell. Berdmore on the Teeth and Gums, page 38, advocates compression by means of lint, agaric, sponge, or cork. Kœcker's Dental Surgery, page 197, recommends compression with cotton dipped in water ascidulated with sulphuric acid.

Lefoulon's Theory and Practice of Dental Surgery, page 181, advocates compression by means of wax, to be retained in its place by the pressure of the jaws, which are to be kept closed by means of a bandage passed around the chin, and fastened to the sinciput; and if these means fail, the actual cautery is the last resource. Jobson's Treatise on the Teeth, page 106, recommends compression by means of lint, cork, or ivory, and the jaws to be brought together by a bandage. Harris' Principles and Practice of Dental Surgery, page 295, says: "Pressure, after all, I believe, is the only thing on which we can rely. If it be so applied, as to act directly upon the mouths of the bleeding vessels, it will be found to be more efficacious than the most powerful styptic, or any other remedy." Professor H. has used compression, by means of lint and sponge saturated with tincture of nut-galls, with much succeess.

Mauray's Treatise on the Dental Art, page 171, recommends compression by means of wax, the jaws to be forcibly closed and retained in position by means of a bandage; also, in extreme cases the actual or potential cautery, but he recommends great caution in the use of the last application.

Fitch's System of Dental Surgery, page 371, recommends compression by means of cotton, in conjunction with styptics, and astringents, such as tincture of galls, a solution of sulphate of copper, a tincture composed of brandy, myrrh and galls, or brandy alone, turpentine, dilute acid, or a solution of the nitrate of silver; and, in some cases the actual cautery. Goddard on the Teeth, p. 113, recommends matico or soldier's weed, solid nitrate of silver pointed somewhat like a pencil and thrust into the alveolus for a few minutes, and the following, which is certainly a valuable stypic: "Cause some alcohol to dissolve as much of the following substances as it is capable of doing, so that it may be a saturated tincture; ergot or secale cornutum, galic acid, then one-fourth of kreosote by measure;" this may be used by saturating lint with it, and plugging up the bleeding cavity; and a watery solution of ergot is also recommended as a local application.

A case by Thomas Embling, Esq., is reported in the 4th vol. of the Am. Journal of Dental Science, page 65, copied from the London Lancet, in which a considerable portion of the alveolar process had been broken off, in the effort to extract a tooth; the hæmorrhage resisted all applications, even lunar caustic, and the actual cautery, it was finally checked by pressure applied by means of the thumb and finger.

Stockton's Dental Intelligencer, vol. 2, page 178, contains a very interesting case, reported by Dr. Roberts, F. R. SS. AC. of Edinburgh, in which the superiority of pressure over all other means, is fully demonstrated; and I refer to the journal for the full particulars of the case.

A case is mentioned in the 2d vol., no. 6, of the New York Dental Recorder, where the hæmorrhage followed the extraction of a tooth, and continued several days, it was finally arrested by the application of a stimulant, to-wit: tincture of anthemis pipethrum, or Spanish pelatory, applied by saturating a pledget of cotton, and filling the cavity with it.

An exceedingly interesting case of obstinate hæmorrhage, following the extraction of a molar tooth, is mentioned in the 8th vol., page 207, of the American Journal of Dental Science, where every application was resisted, until finally, recourse was had to the actual cautery, which fortunately proved successful.

A view of the cases and practice, which we have brought forward clearly shows that the leading remedy for hæmorrhage, as advocated by all the foregoing authorities, is compression; but, from the imperfect manner of producing it, styptics have likewise been generally recommended in conjunction therewith. Lint, cotton, sponge, and linen rag, or any other kinds of simple cloth, are, in my opinion, equally objectionable, because they soon become saturated with blood, and hence, offer no obstruction to its progress, as it would then continue upon the principle of *interstitial circulation.* To the application of wax there are equally strong objections; its want of issimilation to a wet surface, and its structural incapacity to bear a sufficient pressure, when in a plastic state, will cause it to offer but a temporary resistance to the flow of blood between it and the walls of the alveolus; so with plaster of paris, (sulphate of lime,) or even the restoration of the tooth to its socket, the same results will occur as in the application of wax. The *actual cautery* has been confidently recommended as a *last resort.* I can only say of this *heroic remedy, or practice,* that it deserved no consideration from the enlightencd dental profession, and certainly but little comment from myself, except to venture the inquiry, whether any man ever seriously contemplated carrying a *white hot iron* point through a bleeding alveolus to the *patulous mouth* of the bleeding vessel with a view of cauterising it successfully?

Some years ago, I was called to visit an urgent case of alveolar hæmorrhage, where I found a medical gentleman heating a *poker,* with the design of *applying the actual cautery.* I asked him what he thought of *compression?* He replied that it had been already tried, and had failed; and that nothing but the actual cautery would do. I urged compression; and the "*waxed cloth cones*" were applied, and the hæmorrhage was instantly arrested.

The whole course of treatment indicated in alveolar hæmorrhage may be summed up in one word, namely, compression.—But, as there are numerous objections to all the appliances already cited, I have been induced to adopt a plan of treatment not liable to any of the exceptions before taken, and which has been, thus far at least, perfectly successful in every instance in which resort has been made to it. There are many cases in which a bit of lint, or cotton, dipped in any of the ordinary styptics, may arrest alveolar hæmorrhage; but there are also other instances which demand more appropriate appliances, and all the aid which professional skill is able to bring to bear upon them, in order to obtain success; indeed, I have had several cases in the course of my professional career, which *could not have been arrested* by any other local means, than compression upon the plan which I am about to suggest.

The treatment I propose is to arrest hæmorrhage with the "Waxed Cloth Cone," which will be found, I believe, perfectly efficacious in, and applicable to, all cases, not only of a simple, but likewise, of an alarming character.

Explanation of the Drawings.

1. 1. Waxed Cloth Cones of different sizes.
3. The angular strip, which is in progress of being wound into a Cone.
5. Straight line upon which the base of the Cone is formed.
6. Oblique line which forms the slant.
7. Apex of the Cone.
8. Extreme of base.

These angular strips may be cut from one inch to four inches long at the base, and from a quarter of an inch to one inch high on the perpendicular, so as to have cones of all sizes.

The cone is made by dipping fine linen, or cotton cloth, into boiling wax, the cloth is then to be cut into various sizes resembling the diagram, and these are to be rolled into cones. It is now thirteen years since I first constructed these cones in the manner indicated, in order to meet the pressing wants of a case of great emergency, that had resisted every other application, until the experiment in question was crowned with complete success. I can, therefore, with confidence recommend to the profession, after long years of their successful use, that nothing known to the profession of dental surgery will be found more satisfactory in its results. than the foregoing simple, yet efficient remedy.

The cones possess the property of adapting themselves to the varying inequalities of the alveolus; but previous to applying them, they should be immersed, for a moment, in a little warm water, and then, by means of a large blunt plugging instrument, be pressed firmly into the cavity. Some regard should be paid to the size of the cone, as it should approach that of the fang, or fangs, which occupied the alveolar cavities. They will be found to exert positive compression upon the apex and walls of the cavity, without undergoing any change of structure, thereby precluding the possibility of a continuance of the hæmorrhage. The body of the cone being composed of cloth, saturated with a substance impervious to blood, and firmly rolled, precludes the possibility of any moisture becoming insinuated between the folds and disturbing its contact with the alveolus. Examination will demonstrate, that the cone, from its construction and the materials which compose it, is capable of sustaining a great pressure upon its base, and to an extent sufficient to answer all the purposes contemplated in its construction. Previous to the introduction of the cone, the cavity of the alveolus should be wiped out with a lock of cotton, and the mouth well rinsed with cold water; after the cone is forced into the cavity it is to occupy, a compress may be placed over its base, although not always necessary, only in extreme cases, and the patient caused to close his teeth upon it; or, if the patient has no teeth to be antagonized with the compress, it may be increased in its longitudinal dimensions, so as to be in contact with the gum. If the patient should prove refractory, I would recommend Gibson's, or Barton's, bandage, which is used for fracture of the lower jaw.

And in those cases in which there is difficulty in controlling the patient, I would further recommend pinioning the arms, which becomes frequently necessary, in demented persons. It is always the case, that, after preternatural hæmorrhage is fully established, the introduction of any thing causing pressure upon the walls of the alveolar cavity, gives acute pain; this, however, the operator should not regard, but proceed to perform his duty thoroughly. The cone may be removed in twelve, or twenty-four hours, or as soon, as in the judgment of the practitioner, it may be safely done. After the operation of extraction is performed, in all cases it is necessary to caution the patient not to suck the bleeding alveolus. Hæmorrhage, in many instances, of an inordinate character, will be entirely avoided, by close observance of this injunction.

I herewith append a short account, from notes furnished by my friend, C. J. Carpenter, M. D., of this city, of two cases where compression was used in general surgery, with marked success, in controlling hæmorrhage, in his practice. A. B., admitted into the Saint Louis Hospital, 1839, with entire division of the palmar arch by a smooth cut, the hæmorrhage was, in this case, profuse. A compress was laid over the radial artery on the fore arm; the wound was brought together by adhesive straps followed by a light compress; a roller was applied and continued up the limb sufficiently tight to control the hæmorrhage, and yet, not to obstruct the circulation entirely. The bandage was not removed until after the eighth day, at which time the dressing was changed; the wound was found sufficiently healed to allow of the patients discharge.

John Early, aged 35 years, admitted to St. Louis Hospital, in 1839, was laboring under a severe injury of the ankle joint, which had been received some months previously; after consultation with many of our most eminent surgeons, amputation was decided upon, and was performed below the knee. The common circular mode was adopted; no ligatures were used, and hæmorrhage was controlled by compression with the roller; the stump was treated with the usual simple dressings and a compress over all. The patient was placed in bed, the stump elevated, and the tourniquet loosened gradually. The dressings were not removed until the ninth day, when union by the first intention was very nearly effected, and in three weeks from the day of the operation, the patient left the hospital well. No originality is claimed by Dr. Carpenter in the above cases; the practice originated with a German surgeon, who published, some years ago, many cases, which fully illustrated the entire success of the practice of controlling hæmorrhage, by means of compression with the roller, in amputations, &c., &c.

I had the pleasure to assist Dr. C. in this operation, and I must say, that nothing could have been more satisfactory than the operation and its subsequent cure.

In conclusion permit me to say, that the use of the roller as applied to the same purposes to which it now is applied, can be traced back to very remote antiquity; for, in the thirtieth chapter of Ezekiel, 21st verse, we find the following: "Son of man, I have broken the arm of Pharoah, king of Egypt; and lo! it shall not be bound up to be healed, to put a roller to bind it, to make it strong to hold the sword."

## Figures and Tables

**1.1 f1:**