# Errata

**Published:** 1849-01

**Authors:** 


### ERRATA.

*Observations on Hæmorrhage, Vol. ii, No. 1.*

Page 3, line 34, for corner, read cornu.
" 3, " 37, for varine, read ranine.
" 6, " 2, for epistoxis, read epistaxis.
" 9, " 28, ℞ chlorate of potash ʒi.
" 10, " 37, for Le Oran, read Le Dran.
" 12, " 2, for Gaviot, read Gariot.
" 13, " 28, for pipethrum, read pyrethrum.

